# Oxygen-plasma-modified biomimetic nanofibrous scaffolds for enhanced compatibility of cardiovascular implants

**DOI:** 10.3762/bjnano.6.24

**Published:** 2015-01-22

**Authors:** Anna Maria Pappa, Varvara Karagkiozaki, Silke Krol, Spyros Kassavetis, Dimitris Konstantinou, Charalampos Pitsalidis, Lazaros Tzounis, Nikos Pliatsikas, Stergios Logothetidis

**Affiliations:** 1 1Νanomedicine Group, Department of Physics, Lab for “Thin Films Nanosystems & Nanometrology” Aristotle University of Thessaloniki, 54124 Thessaloniki, Greece; 2 Fondazione IRCCS Neurologic Institute “Carlo Besta”, IFOM-IEO-campus, Italy; 3 Department of Applied Physics, Aristotle University of Thessaloniki, 54124 Thessaloniki, Greece

**Keywords:** cardiovascular implants, electrospun nanofibers, plasma treatment, scaffold, tissue engineering

## Abstract

Electrospun nanofibrous scaffolds have been extensively used in several biomedical applications for tissue engineering due to their morphological resemblance to the extracellular matrix (ECM). Especially, there is a need for the cardiovascular implants to exhibit a nanostructured surface that mimics the native endothelium in order to promote endothelialization and to reduce the complications of thrombosis and implant failure. Thus, we herein fabricated poly-ε-caprolactone (PCL) electrospun nanofibrous scaffolds, to serve as coatings for cardiovascular implants and guide tissue regeneration. Oxygen plasma treatment was applied in order to modify the surface chemistry of the scaffold and its effect on cell attachment and growth was evaluated. The conditions of the surface modification were properly adjusted in order to define those conditions of the treatment that result in surfaces favorable for cell growth, while maintaining morphological integrity and mechanical behavior. Goniometry (contact angle measurements), scanning electron microscopy (SEM), atomic force microscopy (AFM), and X-ray photoelectron spectroscopy (XPS) measurements were used to evaluate the morphological and chemical changes induced by the plasma treatment. Moreover, depth-sensing nanoindentation was performed to study the resistance of the plasma-treated scaffolds to plastic deformation. Lastly, the cell studies indicated that all scaffolds were cytocompatible, with the plasma-treated ones expressing a more pronounced cell viability and adhesion. All the above findings demonstrate the great potential of these biomimetic tissue-engineering constructs as efficient coatings for enhanced compatibility of cardiovascular implants.

## Introduction

Cardiovascular diseases represent one of the major causes of mortality in developing countries. For certain clinical cardiovascular applications, implanted devices need to be carefully designed in order to address all the drawbacks related to thrombosis and implant failure [1–2]. To achieve long-term results comparable to those of natural tissues, the ideal material should degrade and remodel with autologous cells into a natural structure [3] while the surface should be able to guide the process of tissue formation. To this end, biomimetic surface coatings and modifications using appropriate durable and biocompatible nanomaterials have already been applied [4–7]. To date, various sophisticated tissue-engineering structures that mimic the extracellular matrix (ECM) have been proposed, which aim to induce the highly desirable in situ endothelialization of vascular biomaterials while minimizing thrombogenicity and inflammation [8–10]. In the case of cardiovascular implants, though, the successful revascularization after implantation remains an unmet clinical problem [11]. This implies that endothelial cells must properly attach and migrate into the surface of the implanted material in order to form an appropriate network that will enable the delivery of blood and nutrients [12–13]. Therefore, the major challenge towards the long-term success of implants, is to find an appropriate surface coating that maintains the intricate balance between promoting in situ endothelialization, while inhibiting thrombosis and other systemic post-surgical complications [14].

Over the last decade, progress in the fundamental research of electrospun nanofibrous scaffolds has accelerated, with a special focus on biomedical applications [15]. This is due to their intrinsic similarities with the ECM of many tissues in the body, which renders them a powerful tool to control cell affinity and adhesion. Moreover, their high surface area to volume ratio, porosity and biodegradability favor cellular interactions, making them ideal candidates for polymer scaffolds [16]. As it is well known, the ideal scaffold should possess good bulk properties in terms of physical and mechanical stability, while the surface should provide high affinity with cells. In order to combine both prerequisites in one biomaterial, a common approach is to use synthetic biomaterials with adequate bulk properties and improve the surface functionalities by applying surface modification treatments [17]. Similarly, polycaprolactone (PCL) represents a commonly used biodegradable synthetic polymer for the fabrication of electrospun nanofibrous scaffolds [18], because of its beneficial bulk properties but lacks the proper surface environment for cellular attachment, mainly due to its strongly hydrophobic character [19]. To date, several surface-engineering techniques have been applied in order to chemically modify surfaces of electrospun nanofibers [9,20–22], including treatments by flame, corona discharge, plasma, photons, electron beam, ion beam, X-rays, and gamma rays. Among them, modifications based on plasma treatment have emerged as a very simple and promising approach to induce surface alterations or even introduce the desired chemical groups onto the surface of a material without affecting its bulk properties [22–23].

The aim of the present study was to effectively modify the surface of nanofibrous PCL scaffolds in order to promote ex situ endothelialization and potentially serve as coatings to enhance the compatibility of cardiovascular implants. The surface of the PCL nanofibrous scaffolds was modified through an oxygen (O_2_)-plasma treatment, in order to increase their surface hydrophilicity by forming oxygen-containing groups at the surface and thus to improve cell adhesion and proliferation. The conditions of the plasma modification were properly adjusted in order to induce the desirable chemical surface changes while maintaining surface integrity and morphology. The applied power of the plasma was selected with respect to its effect on the structural and chemical composition of the scaffold. The untreated and plasma-treated nanofibrous scaffolds were evaluated in terms of surface topography, hydrophilicity, and surface chemistry in order to find the conditions that may lead to improved surface properties without affecting the morphology or the mechanical behaviour of the system. In depth nanoindentation measurements were conducted for the scaffolds under optimal conditions to assess the mechanical performance. MTT assay along with imaging techniques were used to evaluate the influence of the plasma treatment on cell attachment, morphology and viability.

## Results and Discussion

### Morphological characterization of the plasma-treated scaffolds

Electrospinning is a versatile and well-established technique broadly applied for the fabrication of nanofibrous meshes as scaffolds for tissue engineering applications. Electrospun scaffolds exhibit a preferential and easy to modulate morphology due to their unique geometrical features which replicate many in vivo structures [24–25]. The PCL nanofibrous scaffolds, prepared herein through electrospinning, were modified through a treatment with O_2_-plasma using different plasma powers (*P*) of 20 W and 40 W, in order to render their surface more hydrophilic and thus the scaffold more biocompatible. Specifically, the treatment time was kept constant at 5 min, as prolonged time periods of plasma treatment has been already reported to negatively affect the morphology and integrity of the treated systems [26]. A morphological and surface characterization of the treated systems was performed in order to evaluate the optimum treatment conditions and the results are presented below.

**Figure 1:**
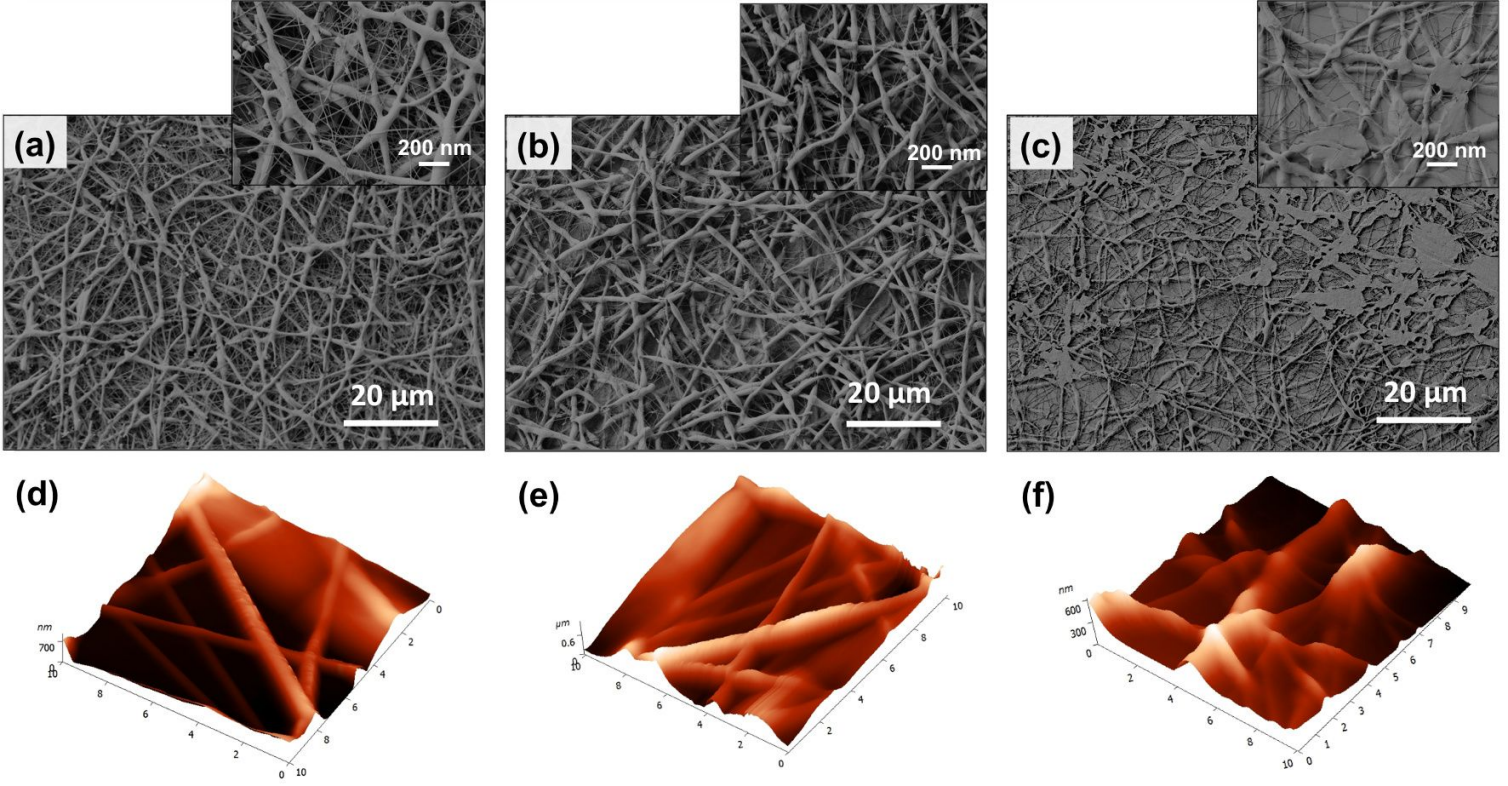
SEM and AFM images of the (a,d) untreated PCL electrospun scaffolds and of the O_2_-plasma modified ones, with (b,e) *P* = 20 W and (c,f) *P* = 40 W (the axis in AFM images is in μm).

The SEM micrographs of the untreated and the plasma-treated electrospun scaffolds are shown in Figure 1a–c. The images clearly indicate the effect of the plasma modification on the structural and surface integrity of the electrospun scaffolds. The unmodified fabricated nanofibers appeared interconnected and randomly stacked in a layer-by-layer configuration, forming pores between the fibers in the non-woven mesh network. It can be observed that the surface of the untreated and the surface-modified nanofibrous scaffolds after applying 20 W power was quite smooth and no morphologically significant differences were found. On the contrary, by increasing the power to 40 W, the plasma effect was very prominent and dramatically affected the morphology of the scaffolds, which resulted in a melting of the fibers as shown in Figure 1c. The mild treatment conditions (high magnification of image in Figure 1b) resulted in the melting of the thinner fibers of the scaffold, which is favorable for the cells, as it provides more space (micropores) between the fibers to elongate and spread [21]. This is highly preferable for certain cell types which exhibit the tendency to spread out and form an elongated cell body, such as the fibroblasts [26], which were incorporated in our study (mouse fibroblast cells-L929).

AFM has been a widely used technique for the observation of composite surfaces on the atomic scale. Plasma-treated surfaces have been effectively studied via AFM due to the ease of sample preparation and excellent resolution [27]. AFM was used in order to determine the topographical features of the studied surfaces and the effect of the plasma treatment on their roughness. The surface roughness parameters of the untreated and plasma-treated electrospun scaffolds are summarized in Table 1, while the obtained topographies of the samples are presented in Figure 1d–f. The average surface roughness (*R*_a_), was found to increase after the O_2_-plasma treatment with mild power conditions (*P* = 20 W) which is attributed to the incorporation of the polar groups on the surface. Indeed, during plasma treatment the polymer chains on the surface break and polar functional groups are created. This leads to an increase in the polarity and the surface energy, resulting in a roughened topography*.* Higher plasma power (*P* = 40 W) significantly decreased *R*_a_ resulting into smoother nanofibrous surfaces compared to the untreated samples, due to the partial polymer melting. These findings, which are in correlation with the morphological changes observed by SEM and AFM imaging, underline the positive effect of the mild power plasma conditions on the nanofibrous scaffolds in terms of surface topography.

**Table 1:** Surface characteristics from AFM analysis and water contact angle measurements for the untreated and the O_2_-plasma-treated nanofibrous scaffolds.

PCL scaffold	roughness, *R*_a_ (nm)	peak-to-valley, *R*_y_ (nm)	contact angle (degrees)

untreated	200.48	225.59	91.3
O_2_, 20 W	245.25	287.22	21.4
O_2_, 40 W	89.5	118.73	19.8

### Chemical characterization of the plasma-treated scaffolds

As can be observed from the contact angle measurements presented in Table 1, the untreated PCL scaffold demonstrated a water contact angle of 91.3°. A significant decrease of the contact angle was found for both modified systems with insignificant differences in the measured values (21.4° and 19.8° for *P* = 20 W and *P* = 40 W, respectively). The insignificant difference in the surface hydrophilicity of the two modified systems, indicates that O_2_-plasma treatment alters the surface hydrophilicity even when applying relatively mild plasma conditions whereas the increase in the power of the plasma treatment does not appear to have any additional effect as far as the goniometry analysis is concerned. It is worth noting that the presented data from the contact angle measurements are particularly valid only for comparative purposes as the nanofibrous scaffolds are not smooth and homogeneous solid surfaces but highly porous structures. Thus, in our case, contact angle measurements are not a reliable technique to gain results concerning the wettability and the surface energy of each system independently [28].

In order to determine the chemical composition of the O_2_-plasma-treated samples as well as the chemical alterations induced after the treatment, XPS measurements were performed. Specifically, high-resolution peak analysis of carbon 1s (C 1s) at the surface was performed for both untreated and plasma-treated PCL scaffolds. According to the obtained XPS data of the C 1s spectra in all the groups of samples three components can be observed, which correspond to the aliphatic carbon bonds (–C–C– or –C–H), carbon single bonded to oxygen (–C–OH or –C–O–), and carbonyl functional groups (O–C=O) located at approximately 285.0, 286.5, and 288.9 eV, respectively.

**Figure 2:**
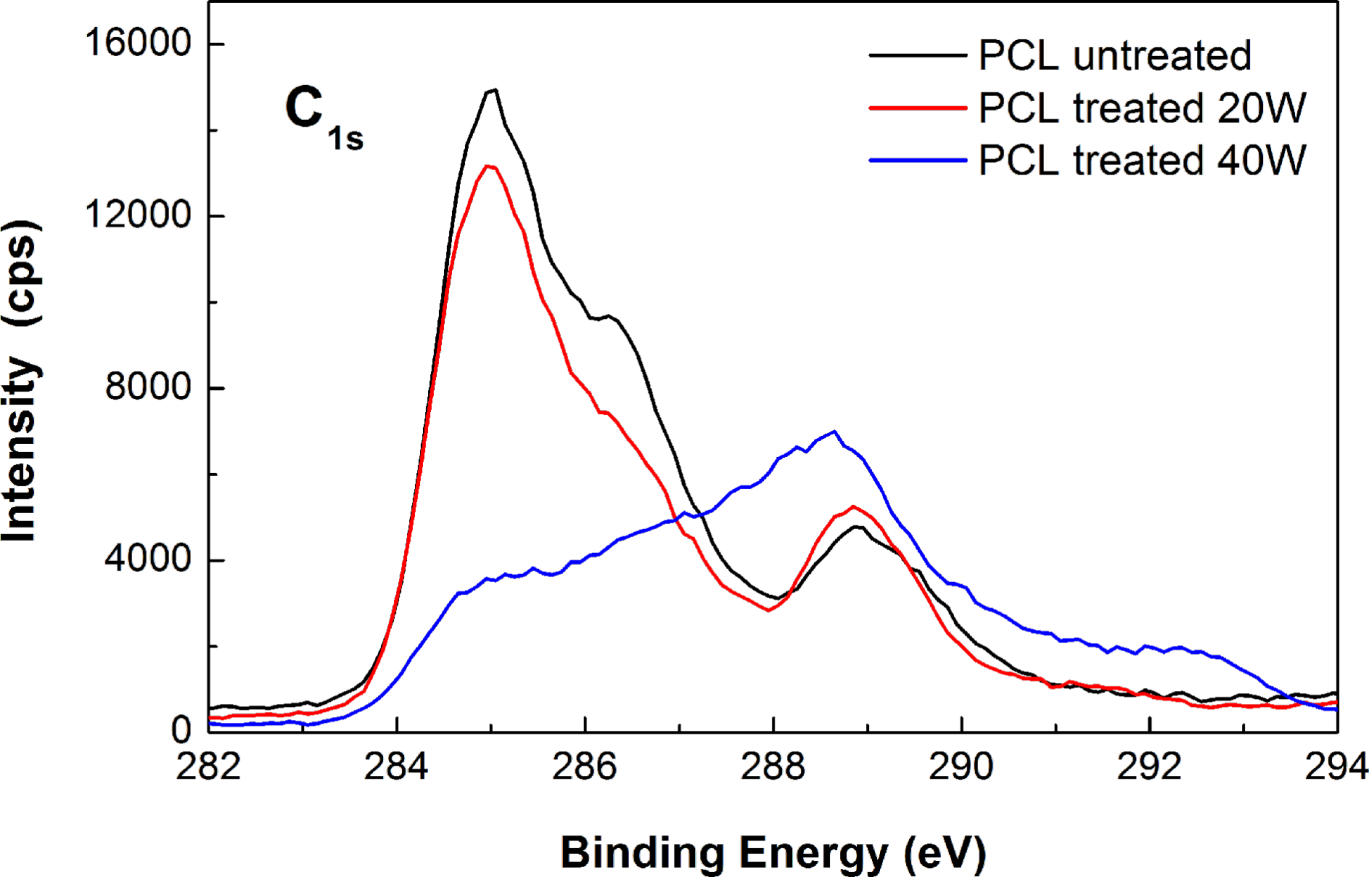
XPS spectra of the untreated PCL electrospun scaffolds and the O_2_-plasma modified ones, with *P* = 20 W and *P* = 40 W.

O_2_-plasma treatment is a commonly used surface modification approach, to introduce oxygen-containing groups onto the surface of a polymer. This leads to an increase in the surface energy of the treated material and therefore enhances its hydrophilic behavior. During this process, the chemical alterations that are induced as a result of the radical reactions between the chain backbone of the polymer and the oxygen in the plasma, modify the surface chemistry, which results in higher numbers of oxygen-containing functional groups. Indeed, changes were observed by the XPS analysis in the intensity of the peaks of the untreated and plasma-treated electrospun PCL scaffolds (Figure 2). This provides evidence of the successful chemical alterations induced by the plasma treatment. These modifications include new oxygen-containing groups formed at the polymer surface, which are evident by the increase in the peak related to the carbonyl functional groups (–C=O). These groups contribute to the improvement of the surface hydrophilicity, which was also confirmed by the goniometry measurements [29]. In Table 2, all the data obtained from the XPS measurements are summarized by means of the surface atomic composition (%) as well as the concentration (%) for the different chemical bonds at the untreated as well as the treated surfaces. Notably, a significant deterioration of the chemical structure was observed in the case of increased plasma power treatment, in which a complete alteration of the C 1s core level peaks is observed. This effect can be presumably attributed to the extensive polymer melting induced by the high power plasma treatment. The significant degradation of the chemical structure of the polymer in line with the deteriorated morphological and topographical characteristics observed through SEM and AFM imaging underline that the milder O_2_-plasma treatment conditions are the most appropriate for enhancing the surface functionality without affecting the morphological properties and chemical structure of the polymer.

**Table 2:** Surface atomic composition (atom %) and concentration of the different chemical bonds (%) on the untreated and the O_2_-plasma-treated nanofibrous scaffolds.

%	untreated	O_2_, 20 W	O_2_, 40 W

C 1s	70.89	69.97	69.91
O 1s	29.11	30.03	30.29
C–C (285.0 eV)	44.2	44.8	13.0
C–O (286.5 eV)	35.0	33.8	22.2
C=O (288.9 eV)	20.8	21.4	48.5

### Mechanical behavior of the plasma-treated scaffolds

A basic principle in regenerative medicine is to maintain the structural integrity of a scaffold after implantation and also to provide the appropriate microstress environment to the cells. It is well known in the implants industry that the mechanical properties of implants should be adequate in order to sustain the stresses and to prevent premature failure [30]. Thus, it is of paramount importance that the coatings developed for implants exhibit a good mechanical performance, which matches with that of the native surrounding tissues. Plasma treatment is known to induce changes onto the outermost layer of a material without interfering with its bulk properties [23]. In order to further confirm this assumption nanomechanical measurements were conducted, in both mildly treated and untreated systems, and the obtained results were compared.

**Figure 3:**
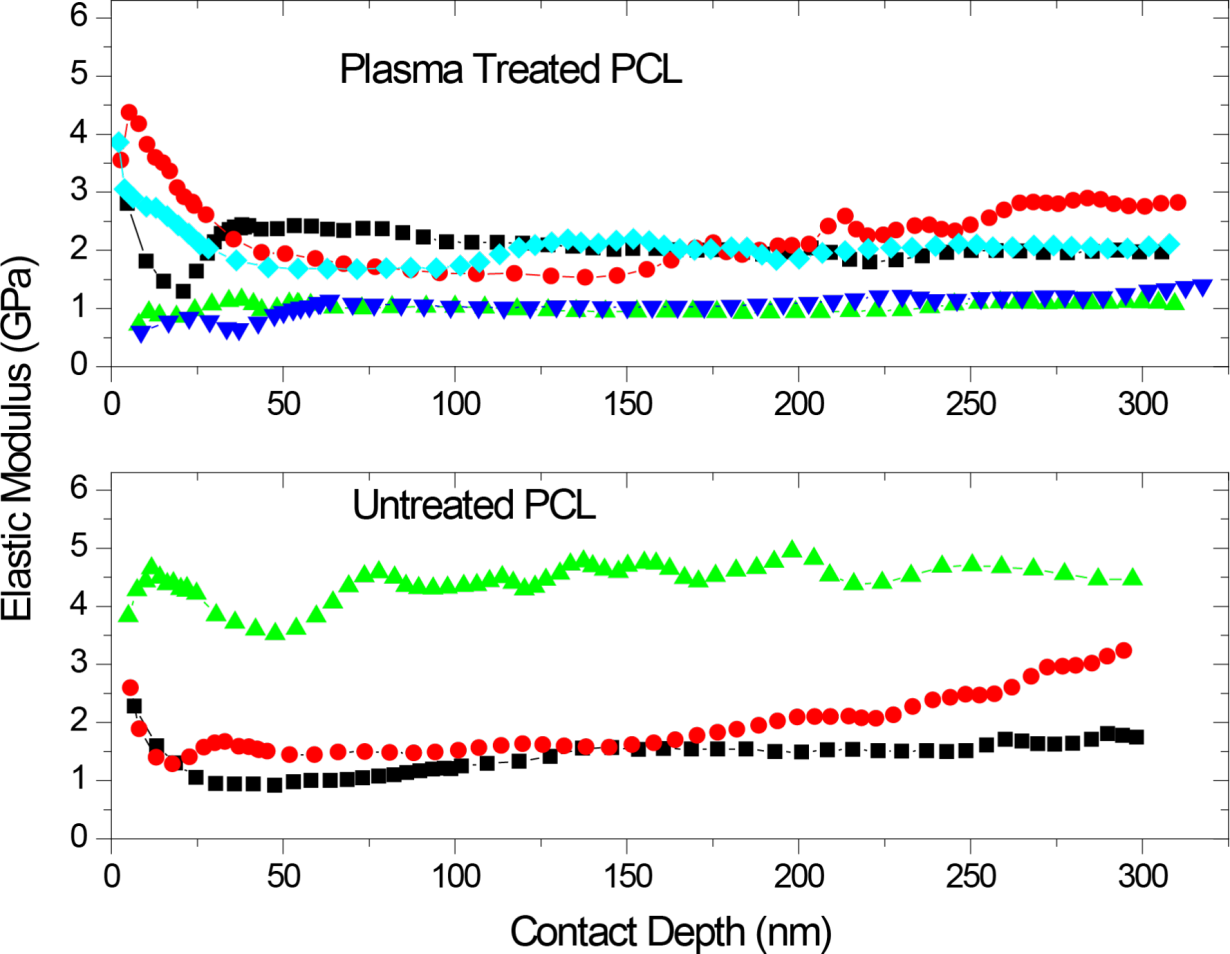
The elastic modulus values of the untreated PCL electrospun scaffolds and the plasma-modified ones (20 W) versus the contact depth. Every curve comes from a nanoindentation measurement to a different surface location of the samples.

The effect of the O_2_-plasma treatment on the mechanical properties of the PCL scaffold was studied by using dynamic nanoindentation. In Figure 3, the elastic modulus (*E*) values of the untreated and the O_2_-plasma-treated PCL versus the contact depth are presented. In every nanoindentation test the same behavior was noticed: As the nanoindenter penetrates from the surface to the ‘body’ of the PCL films the *E* value decreases mainly due to the surface roughness of the PCL, and reaches a plateau after about 50 nm, which corresponds to the *E* of the sample. Therefore, the calculated *E* value, for the untreated and the O_2_-plasma-treated PCL varies between 1–4 GPa and 1.5–2.5 GPa, respectively. Conclusively, the nanoindentation testing showed that the O_2_-plasma did not degrade the mechanical properties of the PCL, in contrary the nanomechanical behavior of the PCL appears to be more homogeneous after the treatment, possibly due to the alternation/homogenization of the PCL surface properties.

### Cellular performance of the plasma-treated scaffolds

As previously mentioned, the main advantage of the electrospun nanofibrous structures in tissue engineering is their morphological resemblance to the ECM, providing the appropriate microenvironment for the cells. Apart from the topographical features, biochemical features are also important to control and favor cell activity. Thus, the ultimate aim of the present work was to modify the hydrophobic surface of electrospun nanofibrous PCL scaffolds, in order to improve the surface chemistry and favor the cell-material interactions without dramatically affecting the morphological features of the material, nor its physicochemical structure. In order to validate the effect of the surface modification on the cell behavior, MTT biological assay in line with imaging techniques were conducted in order to observe the cells viability as well as their morphology and attachment onto the nanofibrous scaffolds.

#### Cell viability assay

**Figure 4:**
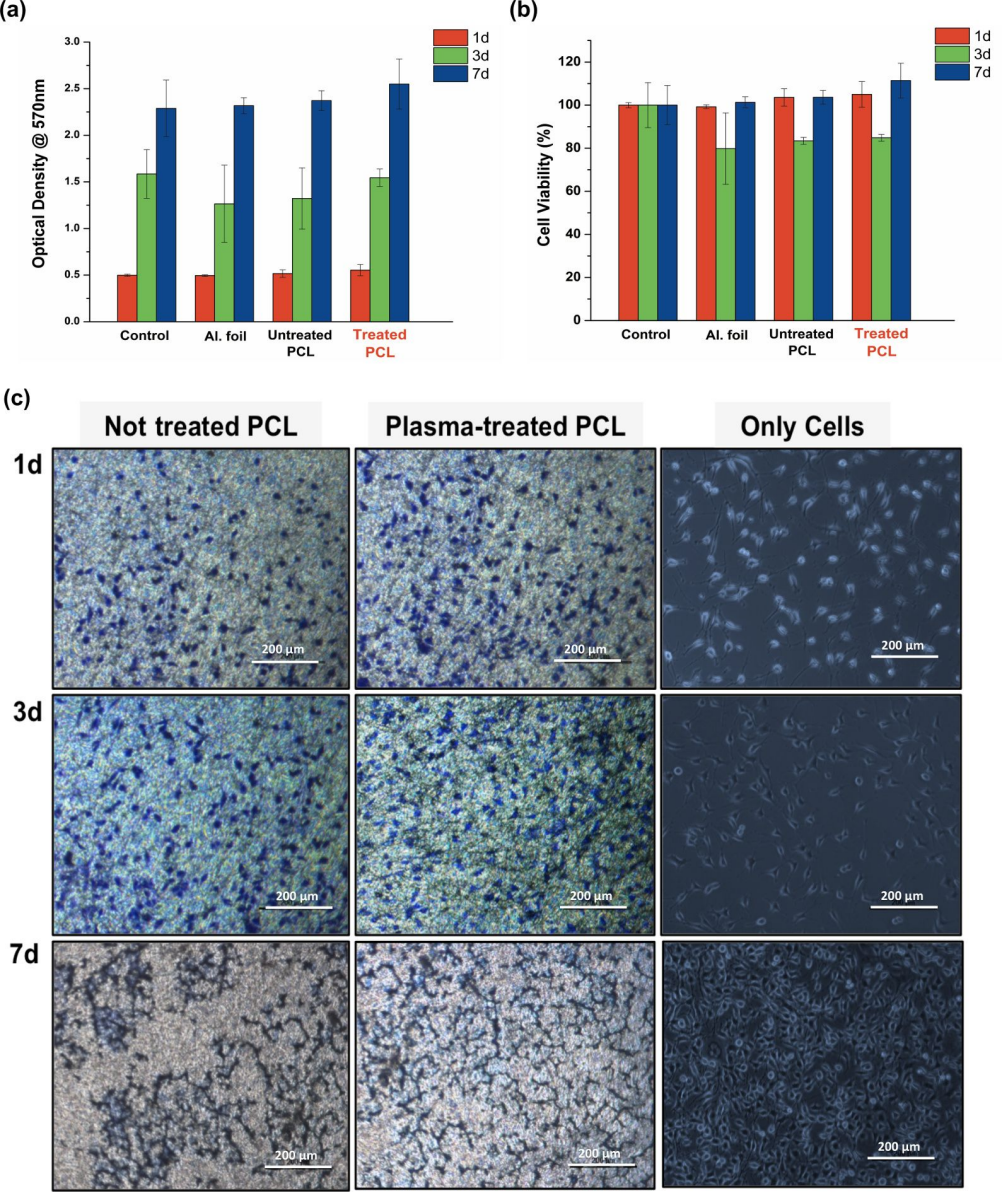
(a,b) MTT assay of L929 cells in direct contact with the examined systems after 1, 3 and 7 days, in terms of optical density values as well as the normalized % cell viability, respectively, and (c) optical imaging of the cell morphology in the predetermined time periods for the untreated and the plasma treated scaffolds.

MTT cell viability assay was performed to the unmodified along with the plasma-treated (*P* = 20 W) scaffold, in order to evaluate the effect of the surface modification on the cellular performance of the fabricated samples in terms of cell viability. The cells used in the present reference study were mouse fibroblasts (L929). In Figure 4a and Figure 4b, the MTT results of the cytotoxicity levels of all the samples (i.e., control group, aluminum foil, untreated scaffold and mildly treated scaffold) in direct contact with the L929s are given. According to the findings, all the fabricated systems were found to be cytocompatible after a period of seven days and exhibited cell viability levels similar to those of the control group. An enhancement in the cell viability (ca. 10%) can be observed in the case of the treated PCL scaffolds after seven days. The improved cellular performance can be attributed to the effective chemical and topographical alterations induced by the plasma treatment, in terms of the increased hydrophilicity as well as surface roughness, respectively. It is noteworthy that in all groups of samples, a general trend in the cell viability, a decrease after three days of culture and an increase afterwards, was observed. This can be attributed to the number of cells that finally managed to adhere onto the desired surfaces, given the fact that cells need time to adapt in new conditions (lag phase). The increase in the cell viability after the three days, which is more apparent in the case of the treated samples indicates the growth and proliferation of the cells in their new microenvironment.

#### Cell adhesion and proliferation

According to Figure 4c, fibroblasts seemed to be securely attached and spread on the surface, regardless of the surface treatments. Interestingly, cells seemed to be attached and spread more on the plasma-treated scaffold surfaces compared to the untreated surfaces, during the first day. A notable enhancement of the spreading is observed in the following days, with higher cell confluency and proliferation in the treated scaffolds compared with the unmodified ones. After seven days of culture, the cells seem to have obtained a spindle-like morphology and especially in the case of the treated scaffolds a notably more uniform spatial distribution of the cells is observed.

**Figure 5:**
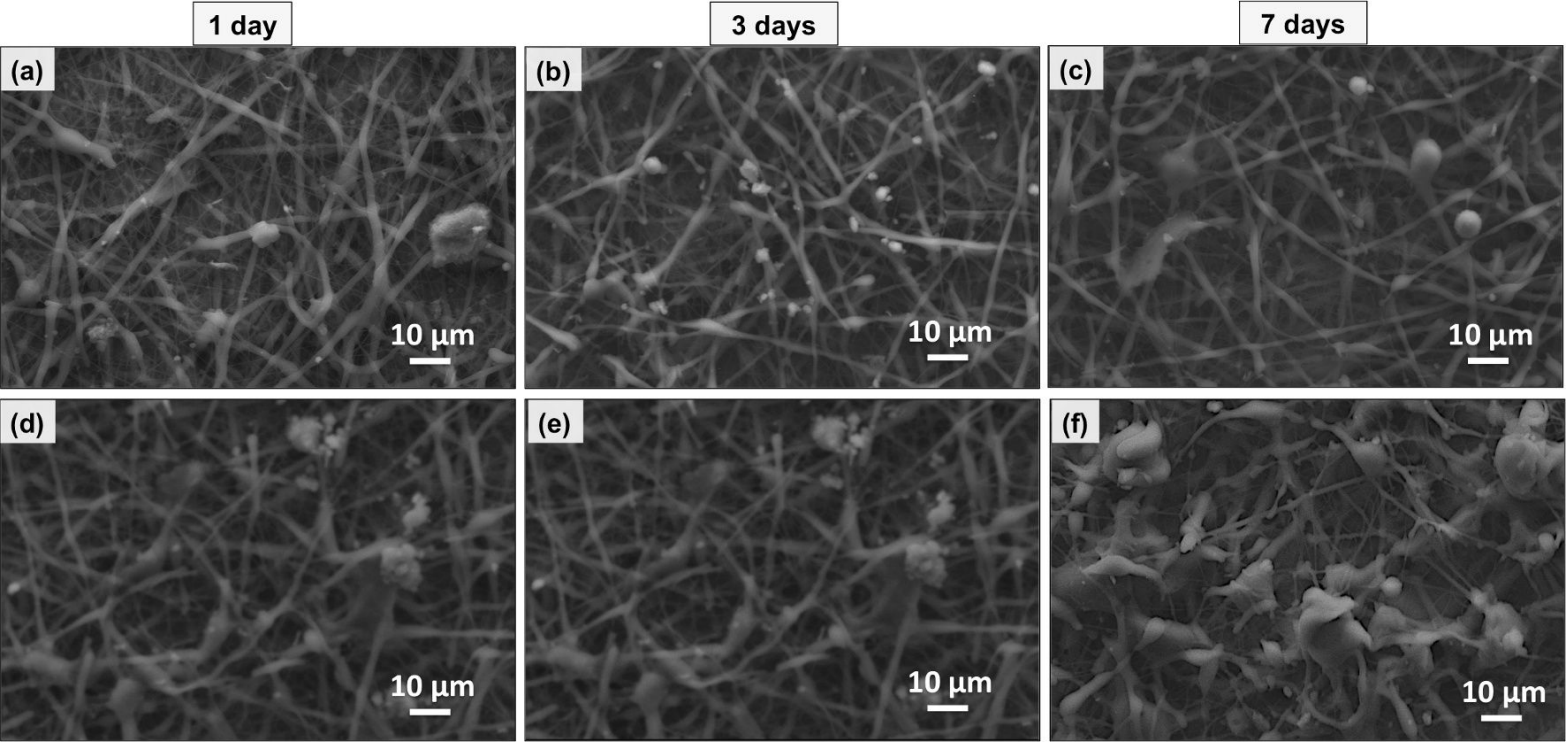
SEM micrographs of the (a–c) untreated PCL electrospun scaffolds for 1,3 and 7 days of direct contact with cells and (d–f) and of the O_2_-plasma-modified scaffolds, at the predetermined time periods.

The morphological analysis through SEM imaging, of the direct contact assay with the L929 fibroblasts for the periods of 1, 3 and 7 days for the untreated and the plasma treated samples is presented in Figure 5. Particularly, a typical spindle- shape cell morphology is observed in the treated samples, while in the untreated surfaces the cells seem to have maintained their round shape. Moreover, the cells seem to be well-dispersed and tightly packed after 7 days in the modified surfaces. The amount of cells is evidently higher in the modified surfaces, underlying the higher affinity of cells the treated surfaces exhibit. After 7 days, a flattened morphology of cells is clearly observed, covering partially the plasma-treated surface. The enhanced cell adhesion and proliferation observed in the modified systems is attributed to plasma-induced chemical alterations of the surface and is in line with the results obtained from the physicochemical characterization of the systems. All the above findings strongly indicate the positive influence of the plasma treatment over the cell adhesion and proliferation.

## Conclusion

Plasma-assisted treatment of the surface of biomaterials has proven to be a straightforward and versatile method for modifying the chemical composition of the surface without affecting its bulk properties. Overall, PCL nanofibrous scaffolds with morphological features that mimic the ECM, and adequate porosity as well as surface hydrophilicity, were successfully fabricated by the electrospinning method. In order to make the scaffold more cytocompatible, oxygen-plasma treatment was used to modify the surface chemistry along with its nanotopography without causing any deterioration of the structure or the integrity of the scaffold and without affecting its mechanical and physical bulk properties. This approach, of controlling the surface properties along with the topographical nanoscale features of the scaffolds could be very useful in the design of novel biomimetic coatings, able to guide tissue regeneration especially in cardiovascular implant industry, where in situ vascular regeneration remains an unmet challenge.

## Experimental

### Scaffold design and fabrication

#### Materials and methods

Polycaprolactone (PCL), *M*_n_ = 45,000 Da, chloroform (≥99.8%), methanol (≥99.9%) were obtained from Sigma (Sigma-Aldrich, Greece). All reagents were used without further purification and all solutions were prepared by deionised water.

The nanofibrous scaffolds were fabricated by electrospinning through the electrostatic spray deposition (ESD) method, (Esprayer ES-2000S, Fuence, Japan) onto aluminum foil and glass substrates. For the fabrication of the polymeric solutions, a solution of PCL was prepared with a total concentration of 20–25 wt % in a solvent mixture of chloroform: methanol (3:1). The solution was electrospun from a 5 mL syringe with a 24 gauge needle and mass flow rate of 10–15 μL/min. A high voltage (15–20 kV) was applied to the tip of the needle when the fluid jet was ejected. The distance between the needle and the moving collector in the XY stage, was set at 40 mm. The glass substrates were cleaned prior to electrospinning with isopropanol and methanol and blow-dried using N_2_ flow. Each fabricated film was left overnight to allow the evaporation of any residual solvents.

#### Plasma treatment

After the fabrication of the electrospun scaffolds, plasma treatment on the nanofibrous scaffolds was performed. The treatment was carried out on a low frequency (40 kHz, 0–200 W) plasma generator (ATTO, Diener Electronic) while using oxygen gas. The procedure was undertaken in a vacuum chamber, which was first evacuated to less than 0.1 mTorr. By supplying oxygen to the chamber the pressure was maintained to 0.25 mTorr while the power was adjusted to up to 40 W for 5 min. Finally, the treated samples were further exposed to the oxygen atmosphere for another 10 min before the sample was taken out from the chamber.

### Characterization techniques

#### Morphological characterization

**SEM:** The morphological analysis of the PCL scaffolds was carried out by using scanning electron microscopy (SEM, NEON40, Carl Zeiss). In order to visualize and evaluate the morphological characteristics of the investigated materials, secondary electron detector was used.

**AFM:** Atomic force microscopy, AFM (AFM Solver, NT-MDT) was used to determine surface topography and roughness of the plasma-treated samples.

#### Chemical characterization

**Contact angle:** Static contact angle measurements using water (Contact angle-surface tensionmeter CAM200, KSV Instruments Ltd) for the untreated and plasma-treated electrospun PCL scaffolds were performed to study and compare the effect of the two treatments on the surface hydrophilicity. At least six different measurements on the plasma-treated surfaces were obtained and the average values for contact angles were calculated. The maximum error in the contact angle measurement did not exceed ±3%.

**XPS:** X-ray photoelectron spectroscopy (XPS) analysis of the surface layers was carried out in an Axis Ultra DLD system by Kratos Analytical using a monochromated Al Kα_1_ X-ray beam as the excitation source. The analyzed area had an elliptical shape with the two axes being about 400 and 700 μm. The pass energy was 160 eV for survey scans and 20 eV for HR spectra. In the latter case, the pass energy would result in a broadening (FWHM) of less than 500 meV for the Ag 3d line. Data interpretation was performed with the Kratos-Vision software.

#### Mechanical characterization

**Nanoindentation:** Dynamic nanoindentation testing (continuous stiffness measurements, Nanoindenter XP) was carried out. A Berkovich type diamond nanoindenter with nominal tip roundness of ca. 50 nm was used to test the samples. Several nanoindents were made to different surface locations of the samples to 300 nm maximum penetration depth. The nanoindentation load–displacement curves were analyzed by using the Oliver–Pharr model to calculate the elastic modulus of the samples versus the indenter penetration (contact) depth [31].

### Cell studies

**MTT assay:** MTT assay (Sigma-Aldrich, Germany) was used to evaluate cell viability. The cells used in this study were mouse fibroblasts L929 and were kindly offered from the Department of Biochemistry of the Aristotle University of Thessaloniki. In a similar manner as described in [32], the cell growth was stopped after 1 day, 3 days and 1 week. After each time point, the cells were incubated with a tenth of the medium of the bromide in 5% CO_2_ (37 °C, 2 h) to allow the formation of water-insoluble formazan crystals. The optical densities (O.D.) of the solutions were read with a spectrophotometer, at the wavelength of 570 nm with respect to the reference wavelength of 690 nm. Data (*n* = 3) were presented as means of O.D. values as well as normalized according to the control and presented as % cell viability.

**Optical imaging through methylene blue staining**: Once fibroblasts were seeded onto either unmodified or surface-modified nanofibrous scaffolds, cellular morphology and cell attachment were observed after 1, 3 and 7 days. Cells were fixed, after the predetermined time periods, in 4% formaldehyde/PBS, at rt for 20 min, permeabilized with PBS and incubated with methylene blue (blue fluorescence) at rt for 30 min. The cell surface was observed with an optical microscope (Carl Zeiss, Germany).
